# Clinical characteristics and etiological analysis in liver abscess: a retrospective study

**DOI:** 10.3389/fcimb.2026.1850104

**Published:** 2026-07-23

**Authors:** Liru Zhao, Meng Zhou, Dongbo Li, Zhongle Cheng

**Affiliations:** The First Affiliated Hospital of Anhui Medical University, Hefei, Anhui, China

**Keywords:** antimicrobial resistance, clinical features, *Escherichia coli*, *Klebsiella pneumoniae*, liver abscess

## Abstract

**Introduction:**

This study aimed to investigate the distribution and antimicrobial resistance patterns of pathogens isolated from patients with liver abscess, and to correlate these findings with clinical characteristics, providing evidence for empirical antibiotic therapy.

**Methods:**

A retrospective analysis of bacterial pathogen distribution, antimicrobial susceptibility patterns, and clinical characteristics in patients with liver abscess at a tertiary hospital was conducted from April 2019 to March 2025. Clinical and microbiological data were extracted from medical records. According to culture results, patients were classified into the culture-negative liver abscess (CNLA) and culture-positive liver abscess (CPLA) groups. The CPLA cases were further divided into Klebsiella pneumoniae liver abscess (KPLA), Escherichia coli liver abscess (ECLA), and other pathogen groups.

**Results:**

Multivariable logistic regression analysis showed that diabetes, malignant tumors, and hepatic insufficiency were independently associated with CPLA. Furthermore, diabetes was independently associated with KPLA, whereas malignant tumors were negatively associated with KPLA. Among the 297 CPLA patients, 326 pathogenic isolates were identified, including 271 patients with monomicrobial infections and 26 patients with polymicrobial infections. Klebsiella pneumoniae was the most frequently isolated pathogen (73.5%), followed by Escherichia coli (14.9%). Antimicrobial resistance pattern analysis revealed that 22.4% of isolates were multidrug-resistant, 10.4% were extended-spectrum b-lactamase (ESBL) producers, and 3.7% were carbapenem-resistant Enterobacterales (CRE).

**Discussion:**

CPLA and CNLA patients exhibited different clinical characteristics, with the latter posing a greater challenge to empirical therapy due to the lack of microbiological evidence. KPLA was more commonly associated with diabetes and generally maintained susceptibility to commonly used antimicrobial agents, whereas ECLA was more frequently associated with malignancy and biliary tract disease and demonstrated a higher antimicrobial resistance burden. For patients without microbiological evidence or with culture-negative results, empirical therapy should prioritize coverage of Enterobacterales, with treatment optimization based on subsequent susceptibility testing results.

## 1 Introduction

Liver abscess (LA) refers to a pyogenic infection of the liver caused by the invasion of pathogenic microorganisms. It is a common severe infectious disease in clinical settings, and its incidence has been rising in recent years (Sharma et al., 2018). This increasing incidence may be partly related to population aging, the rising prevalence of chronic diseases such as diabetes, and improved detection due to advances in modern imaging techniques (Meddings et al., 2010; Yin et al., 2021; Yoo et al., 2021).

Common pathogens causing liver abscesses include bacteria, fungi, and amoebae. Among them, pyogenic liver abscess (PLA) is the most common type. In recent years, the predominant isolated pathogen has shifted from *Escherichia coli* (*E. coli*) to *Klebsiella pneumoniae* (*K. pneumoniae)* (Long et al., 2024). Previous studies have suggested that this change may be partly associated with the emergence and dissemination of *K. pneumoniae* strains, particularly those related to the K1 capsular serotype (Chuang et al., 2006; Chung et al., 2007; Yeh et al., 2007; Chung et al., 2008).

Moreover, patients with liver abscess often have underlying diseases such as diabetes and malignant tumors (Thomsen et al., 2007; Wang et al., 2023; Wendt et al., 2024). These two pathogens differ in their pathogenic mechanisms, host predispositions, and antimicrobial resistance patterns: *K. pneumoniae* liver abscess often occurs in patients with diabetes, whereas *E. coli* liver abscess is more commonly secondary to biliary tract disease (Chen et al., 2007; Shelat et al., 2015; Qian et al., 2016).

With the widespread use of antimicrobial agents, antimicrobial resistance remains an important concern in the management of liver abscess (Liu and Wang, 2025). Previous studies have reported liver abscess cases associated with carbapenem-resistant *K. pneumoniae* (CRKP), while antimicrobial resistance in *E. coli* also remains a prominent issue (Lee et al., 2017; Chen et al., 2022; Liu et al., 2024). Because pathogen distribution and antimicrobial resistance patterns may vary across regions and bacterial species, local microbiological and antimicrobial susceptibility data are important for empirical antimicrobial treatment decisions.

Meanwhile, pathogen identification and antimicrobial susceptibility testing provide an important basis for pathogen-directed therapy. In clinical practice, however, despite advances in microbiological diagnostic techniques, a proportion of liver abscess cases remain culture-negative, which limits pathogen-directed therapy and necessitates empirical antimicrobial treatment. Therefore, evaluating the clinical characteristics of culture-positive liver abscess (CPLA) and culture-negative liver abscess (CNLA), together with local pathogen distribution and antimicrobial susceptibility patterns, may provide useful evidence for clinical management and rational antimicrobial use.

Therefore, this study aimed to characterize liver abscesses from both culture-based classification (CPLA versus CNLA) and pathogen-based classification (KPLA versus ECLA), and further analyze the pathogen distribution characteristics and antimicrobial resistance patterns of major pathogens, thereby providing evidence for clinical practice, early pathogen identification, and optimization of antibacterial treatment strategies.

## 2 Materials and methods

### 2.1 Study design

The data were derived from the database of liver abscess cases admitted to the First Affiliated Hospital of Anhui Medical University from April 2019 to March 2025. The diagnostic criteria for liver abscess are as follows: (1) There are typical clinical symptoms of liver abscess, such as fever and abdominal pain. (2) Radiological evidence on ultrasonography, computed tomography(CT), or magnetic resonance imaging (MRI) compatible with liver abscess. Radiological findings suggestive of liver abscess included single or multiple intrahepatic cystic or liquefactive lesions with thick or irregular walls, internal echoes or debris on ultrasonography, heterogeneous attenuation or signal intensity, gas formation or air–fluid levels, and peripheral or rim enhancement after contrast administration. On MRI, typical findings included low signal intensity on T1-weighted images and high signal intensity on T2-weighted images.

Microbiological examination was required for study inclusion, but microbiological positivity was not a mandatory inclusion criterion. Blood cultures were performed for all enrolled patients, but not all patients underwent abscess culture. Available abscess culture results were obtained from abscess fluid specimens collected during drainage procedures. In our institution, ultrasound-guided percutaneous drainage was performed when clinically indicated. After local anesthesia, a drainage catheter was inserted into the abscess cavity under real-time ultrasonographic guidance, and abscess fluid specimens were collected for bacterial culture and antimicrobial susceptibility testing.

**Figure 1:**
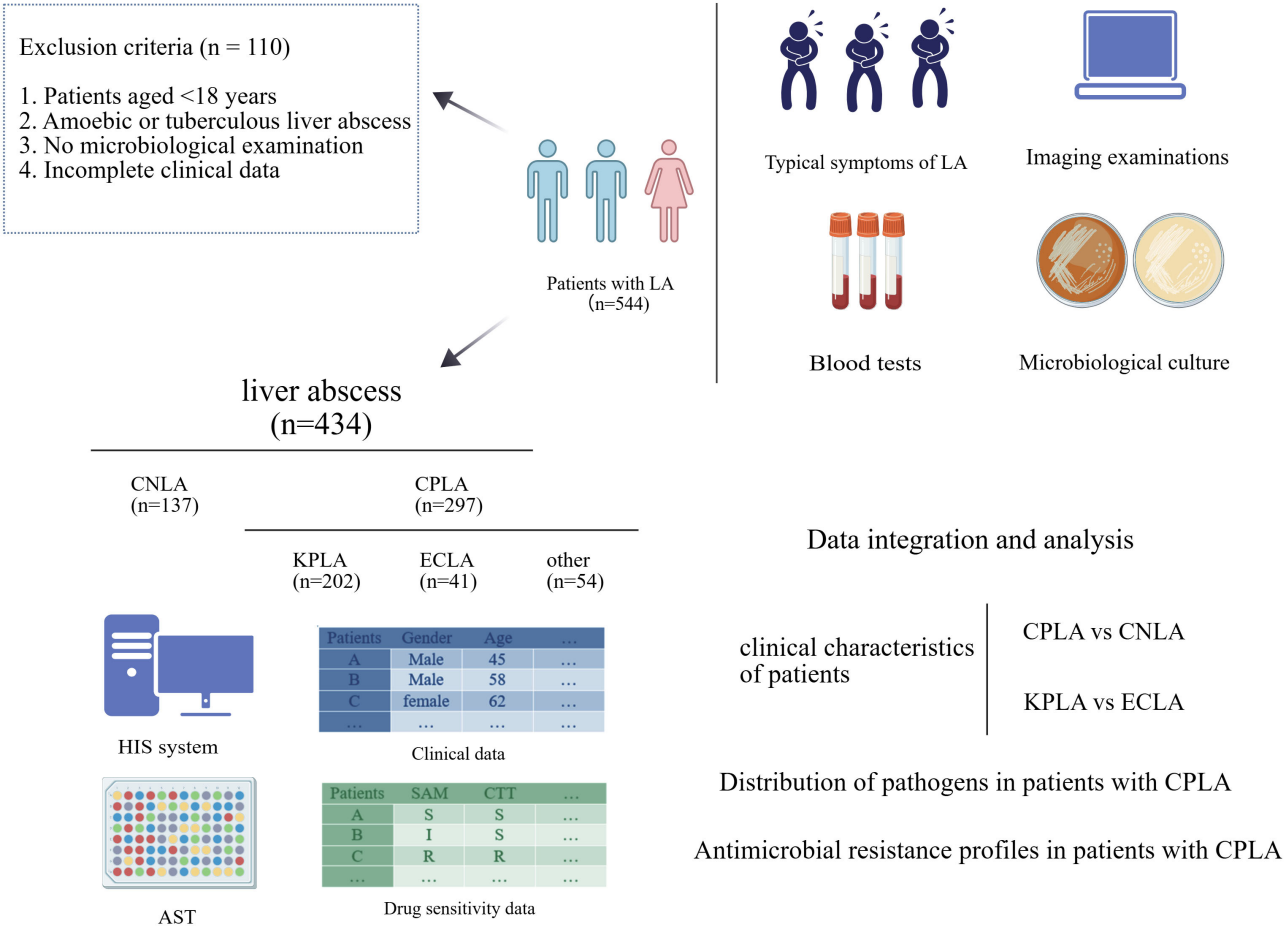
Schematic overview of the study design created with BioGDP.com (Jiang et al., 2025).

Patients with negative blood and abscess culture results were classified as culture-negative liver abscess (CNLA), whereas those with positive culture results from either blood or abscess culture were classified as culture-positive liver abscess (CPLA). Potential contaminants were evaluated according to the available clinical evidence; organisms without supportive evidence for true infection were regarded as contaminants and were not considered causative pathogens. The exclusion criteria are: (1) Patients aged <18 years, (2) Amoebic or tuberculous liver abscess. (3) No microbiological examination. (4) Information is incomplete. The study design is shown in **Figure 1** (Jiang et al., 2025).

### 2.2 Data collection

The data were collected from the patient’s medical records in the hospital’s electronic medical system. The data included demographic characteristics (gender and age), microbiological tests (bacterial species diagnosis and drug sensitivity results), comorbidities (diabetes, hypertension, malignant tumors, sepsis, cerebral infarction, hepatic insufficiency, kidney disease, pulmonary disease, biliary tract disease), whether critical conditions occurred, admission route, hospital-related factors (length of hospital stay, surgical procedures, treatment), and discharge outcomes (improvement, cure, and death). Patients with unclear discharge outcomes, including transfer to other departments or institutions, or discharge against medical advice, were excluded from discharge outcome analysis. Critical illness during hospitalization was defined as a life-threatening clinical condition determined by the treating physician based on a comprehensive assessment of the patient’s underlying comorbidities, vital signs, and the presence of emergent complications.

### 2.3 Pathogen identification and antimicrobial susceptibility testing

Blood and pus specimens were collected aseptically by clinicians and submitted to the clinical microbiology laboratory for culture and identification. Pus specimens were inoculated onto appropriate culture media and incubated at 37 °C for 24–48 h. Blood specimens were inoculated into blood culture bottles and incubated using an automated blood culture system. After a positive signal was detected, positive blood culture samples were subcultured onto appropriate culture media and incubated at 37 °C for 24–48 h. After positive growth, pathogen identification was performed using MALDI-TOF MS.

Antimicrobial susceptibility testing was performed using the VITEK 2 Compact system and the disk diffusion (Kirby–Bauer) method. The interpretation of the drug sensitivity results is based on the CLSI M100 34th edition guidelines.

Multidrug-resistant (MDR) bacteria were defined as non-susceptible to at least one agent in three or more antimicrobial categories (Magiorakos et al., ; Tamma et al., 2024). Polymicrobial infections are defined as culturing ≥ 2 types of pathogenic bacteria from the patient’s specimen; when different organisms were isolated from different specimen sources, the case was classified as polymicrobial infection only if all organisms were considered clinically significant pathogens.

Carrying ESBL pathogenic bacteria means that the bacteria produce extended-spectrum β-lactamases, which can hydrolyze antibiotics such as penicillins, cephalosporins, and monobactams. ESBL production was determined using the combination disk confirmatory test. An increase in the inhibition zone diameter of ≥5 mm for the cefotaxime/clavulanic acid disk compared with the cefotaxime disk alone was interpreted as ESBL-positive.

### 2.4 Statistical analysis

Statistical analyses were performed using SPSS statistical software (version 26.0; IBM Corp, Armonk, NY, USA). Continuous variables were tested for normality using the Shapiro-Wilk test. Normally distributed variables were expressed as mean ± standard deviation and compared using Student’s t-test, whereas non-normally distributed variables were expressed as median (interquartile range, IQR) and compared using the Mann-Whitney U test. Categorical variables were expressed as numbers (percentages) and compared using Pearson’s X² test or Fisher’s exact test, as appropriate. Variables with *P <*0.05 in univariate analysis were included in multivariable logistic regression analysis to identify independent risk factors. All statistical tests were two-sided, and *P <*0.05 was considered statistically significant.

### 2.5 Ethics statement

This study was conducted in accordance with the Helsinki Declaration, and the protocol was approved by the Ethics Committee of the First Affiliated Hospital of Anhui Medical University (approval no. 2026-03-89). Informed Consent Statement: Due to the retrospective nature of the study, the requirement for informed consent was waived by the institutional review board.

## 3 Results

### 3.1 Clinical characteristics of patients with CPLA and CNLA

A total of 544 patients with liver abscess were screened, of whom 434 patients were finally included in the study, including patients with CPLA (n=297, 68.4%) and CNLA (n=137, 31.6%). The clinical characteristics of patients with CPLA and CNLA are summarized in **Table 1**. Compared with CNLA patients, patients with CPLA had a longer length of hospital stay [18 (12-26) vs. 15 (11-19), *P* = 0.001], and higher rates of sepsis, diabetes, hepatic insufficiency, malignant tumors, and pulmonary disease(all *P* < 0.05), as well as higher severity of illness during hospitalization. There were no significant differences in age, sex, or discharge outcome between the two groups. The multivariable logistic regression analysis results are shown in **Table 2**. Diabetes (OR 2.002, 95% CI 1.273–3.150, *P* = 0.003), malignant tumors (OR 3.128, 95% CI 1.535–6.375, *P* = 0.002), and hepatic insufficiency (OR 3.216, 95% CI 1.391–7.433, *P* = 0.006) were independently associated with culture positivity. Neither age (OR 0.999, 95% CI 0.984–1.014, *P* = 0.894) nor gender (OR 1.353, 95% CI 0.871–2.102, *P* = 0.179) was significantly associated with culture positivity.

**Table 1:** Comparison of clinical characteristics between patients with CPLA and CNLA.

Variables	CPLA (n=297)	CNLA (n=137)	*P* value
Age,(years) (median, IQR)	59 (52-70)	59 (53-71)	0.887
Gender, n(%)			
Male	205(69.0)	83(60.6)	0.084
Female	92(31.0)	54(39.4)	
Admission route, n(%)			
Outpatient	51(17.2)	26(19)	0.647
Emergency department	246(82.8)	111(81)	
Length of hospital stay, (days)(median, IQR)	18 (12-26)	15 (11-19)	0.001
Comorbidities, n(%)			
Sepsis	90(30.3)	6(4.4)	<0.001
metastatic abscesses	17(5.7)	4(2.9)	0.206
Diabetes	129(43.4)	40(29.2)	0.005
Hypertension	66(22.2)	31(22.6)	0.925
Cerebral infarction	28(9.4)	9(6.6)	0.322
Hepatic-insufficiency	47(15.8)	7(5)	0.002
Malignant tumors	52(17.5)	11(8)	0.009
Pulmonary disease	107(36.0)	35(25.5)	0.031
Biliary tract disease	87(29.3)	33(24.1)	0.260
Renal disease	61(20.5)	30(21.9)	0.746
Treatment method, n(%)			
Puncture and drainage	193(65.0)	92(67.2)	0.658
supportive therapy	37(12.5)	16(11.7)	0.818
Intravenous antibiotic therapy	86(29.0)	32(23.4)	0.223
Critical illness occurrence, n(%)			
Yes	141(47.5)	50(36.5)	0.032
No	156(52.5)	87(63.5)	
ICU admission, n(%)			
Yes	14(4.7)	2(1.5)	0.095
No	283(95.3)	135(98.5)	
Discharge condition, n(%)			
Cured	27(9.1)	14(10.2)	0.709
Improved	233(78.5)	111(81)	0.706
Death	2(1)	1(1)	1

**Table 2:** Multivariable logistic regression analyses of factors associated with culture positivity.

CPLA vs CNLA	*P* value	OR (95% CI)
Age(years)	0.894	0.999(0.984-1.014)
Gender	0.179	1.353(0.871-2.102)
Diabetes	0.003	2.002(1.273-3.150)
Malignant tumors	0.002	3.128(1.535-6.375)
Hepatic-insufficiency	0.006	3.216(1.391-7.433)

### 3.2 Clinical characteristics of patients with KPLA and ECLA

*K. pneumoniae* and *E. coli* were the most frequently isolated pathogens in this study. The clinical characteristics of patients with KPLA and ECLA are summarized in **Table 3**. Compared with ECLA patients, patients with KPLA were younger[58.0 (51.0-67.8) vs. 66.0 (58.0-73.0), *P<* 0.001], had a higher prevalence of diabetes(*P* = 0.002), and underwent puncture drainage more frequently(*P* = 0.009). In contrast, malignant tumors(*P<* 0.001) and biliary tract diseases(*P* = 0.001) were more common in patients with ECLA. There were no significant differences in gender, length of hospital stay, or discharge outcome between the two groups. The multivariable logistic regression analysis results are shown in **Table 4**. Diabetes (OR 2.613, 95% CI 1.155–5.911, *P* = 0.021) and younger age (OR 0.966, 95% CI 0.938–0.995, *P* = 0.022) were independently associated with KPLA, whereas malignant tumors (OR 0.303,95% CI 0.127-0.722, *P* = 0.007) were negatively associated with KPLA. Biliary tract disease (OR 0.481, 95% CI 0.225–1.027, *P* = 0.058) was marginally associated with KPLA. No significant association was observed between gender (OR 1.199, 95% CI 0.588-2.443, *P* = 0.618) and KPLA.

**Table 3:** Comparison of clinical characteristics between patients with KPLA and ECLA.

Variables	KPLA (n=202)	ECLA (n=41)	*P* value
Age, years, median (IQR)	58.0 (51.0-67.8)	66.0 (58.0-73.0)	<0.001
Gender, n(%)			
Male	141(69.8)	27 (65.9)	0.618
Female	61(30.2)	14 (34.1)	
Admission route, n (%)			
Outpatient	163(80.7)	35 (85.4)	0.483
Emergency department	39(19.3)	6 (14.6)	
Length of hospital stay, days, median (IQR)	17.5 (12.0-25.8)	25.0 (22.0-26.0)	0.355
Comorbidities, n (%)			
Sepsis	71(35.1)	12 (29.3)	0.363
metastatic abscesses	12(5.9)	3 (7.3)	0.738
Diabetes	108(53.5)	11 (26.8)	0.002
Hypertension	52(25.7)	8 (19.5)	0.399
Cerebral infarction	22(10.9)	5 (12.2)	0.809
Hepatic insufficiency	31(15.3)	4 (9.8)	0.317
Malignant tumors	14(6.9)	14 (34.1)	<0.001
Pulmonary disease	48(23.8)	12 (29.3)	0.127
Biliary tract disease	46(22.8)	20 (48.8)	0.001
Renal disease	24(11.9)	9 (22)	0.643
Treatment method, n (%)			
Puncture and drainage	141(69.8)	20 (48.8)	0.009
supportive therapy	25(12.4)	6 (14.6)	0.693
Intravenous antibiotic therapy	60(29.7)	12 (29.3)	0.956
Critical illness occurrence, n (%)			
Yes	97(48	20 (48.8)	0.929
No	105 (52)	21 (51.2)	
ICU admission, n (%)			
Yes	11 (5.4)	1 (2.4)	0.418
No	191 (94.6)	40 (97.6)	
Discharge outcome, n (%)			
Cured	18 (8.9)	4 (9.8)	0.864
Improved	169 (83.7)	33 (80.5)	0.621
Death	1 (0.1)	0 (0)	1

**Table 4:** Multivariable logistic regression analyses of factors associated with and KPLA.

KPLA vs ECLA	P value	OR (95% CI)
Age(years)	0.022	0.966(0.938-0.995)
Gender	0.618	1.199(0.588-2.443)
Diabetes	0.021	2.613(1.155-5.911)
Malignant tumors	0.007	0.303(0.127-0.722)
Biliary tract disease	0.058	0.481(0.225-1.027)

### 3.3 Distribution of pathogens in patients with CPLA

In this study, 326 pathogens were isolated from patients with CPLA(n=297). Most patients (91.2%, n=271) had monomicrobial infections, while a minority (7.4%, n=22) had dual-pathogen infections. Only a very small number (1.4%, n=4) had triple-pathogen infections. Gram-negative bacteria (84.4%, n=275) were the most common, followed by Gram-positive bacteria (12.9%, n=42) and fungi (2.7%, n=9).

Among the 26 patients with polymicrobial infection, the microbiological composition is summarized in **Table 5**. The most common composition was Gram-negative bacteria plus *Enterococcus* spp. (8/26, 30.8%), followed by multiple Gram-negative bacteria (7/26, 26.9%) and fungi plus Gram-negative bacteria (4/26, 15.4%).

**Table 5:** Combinations of pathogens in polymicrobial infections.

Microbiological composition	No. of cases, n (%)	Representative combinations
Gram-negative bacteria *+ Staphylococcus* spp.	2 (7.7%)	*Escherichia coli* + *Staphylococcus* spp. (n=1) *Klebsiella pneumoniae* + *Staphylococcus* spp. (n=1)
Gram-negative bacteria *+ Streptococcus* spp.	3 (11.6%)	*Pseudomonas aeruginosa* + *Streptococcus* spp. (n=1) *Escherichia coli* + *Streptococcus* spp. (n=1) *Klebsiella pneumoniae* + *Streptococcus* spp. (n=1)
Gram-negative bacteria *+ Enterococcus* spp.	8 (30.8%)	*Escherichia coli* + *Enterococcus* spp. (n=4) *Klebsiella pneumoniae* + *Enterococcus* spp. (n=1) *Escherichia coli* + *Serratia marcescens* + *Enterococcus* spp. (n=1) *Escherichia coli* + *Morganella morganii* + *Enterococcus* spp. (n=1) *Klebsiella pneumoniae* + *Pseudomonas aeruginosa* + *Enterococcus* spp. (n=1)
Multiple Gram-negative bacteria	7 (26.9%)	*Citrobacter braakii*+ *Pseudomonas aeruginosa* (n=1) *Klebsiella pneumoniae*+*Morganella morganii* (n=1) *Escherichia coli*+ *Proteus* spp. (n=2) *Acinetobacter junii*+*Acinetobacter caviae* (n=1) *Klebsiella pneumoniae*+*Pseudomonas aeruginosa* + *Klebsiella oxytoca* (n=1) *Klebsiella pneumoniae*+*Acinetobacter baumannii* (n=1)
Multiple Gram-positive bacteria	1 (3.8%)	*Enterococcus* spp. + *Streptococcus* spp. (n=1)
Gram-positive bacteria + Fungi	1(3.8%)	*Enterococcus* spp. + *Candida tropicalis* (n=1)
Gram-negative bacteria + Fungi	4(15.4%)	*Klebsiella pneumoniae* + *Candida albicans* (n=1) *Pseudomonas aeruginosa* + *Candida glabrata* (n=1) *Escherichia coli* + *Candida glabrata* (n=2)

**Figure 2:**
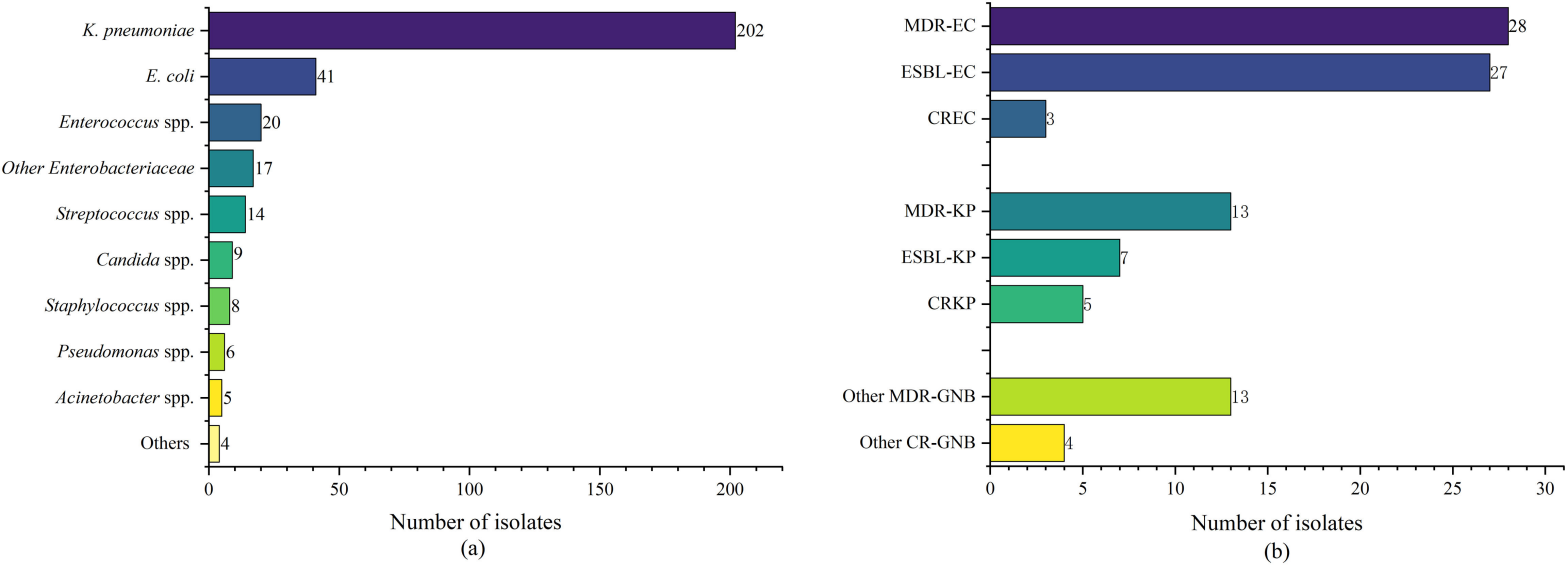
Distribution of pathogens in CPLA and major antimicrobial-resistant phenotypes among liver abscess isolates. **(a)** Distribution of pathogens isolated from liver abscess patients. **(b)** Distribution of multidrug-resistant (MDR), carbapenem-resistant (CR), and extended-spectrum β-lactamase-producing (ESBL) isolates among major pathogens.

The most common pathogen isolated from patients with CPLA was *K. pneumoniae* (73.5%, n=202), followed by *E. coli*(14.9%, n=41). Among Gram-positive bacteria, *Enterococcus faecium* was the most common(31%, n=13). Fungi were relatively rare. The distribution of isolated pathogens is shown in **Figure 2a**.

### 3.4 Antimicrobial resistance profiles in patients with CPLA

Among the 326 isolates, multidrug-resistant bacteria accounted for 22.4%(n=73), ESBL-producing bacteria accounted for 10.4%(n=34), and ESBL-producing *E. coli* was significantly more prevalent than ESBL-producing *K. pneumoniae* (65.9% vs 3.5%, *P<*0.001). Carbapenem-resistant bacteria were uncommon (3.7%,n=12). Methicillin-resistant *Staphylococcus aureus* (MRSA) or vancomycin-resistant *Enterococcus* (VRE) was not detected. The distribution of multidrug-resistant, ESBL-producing, and carbapenem-resistant isolates is shown in **Figure 2b**.

**Figure 3:**
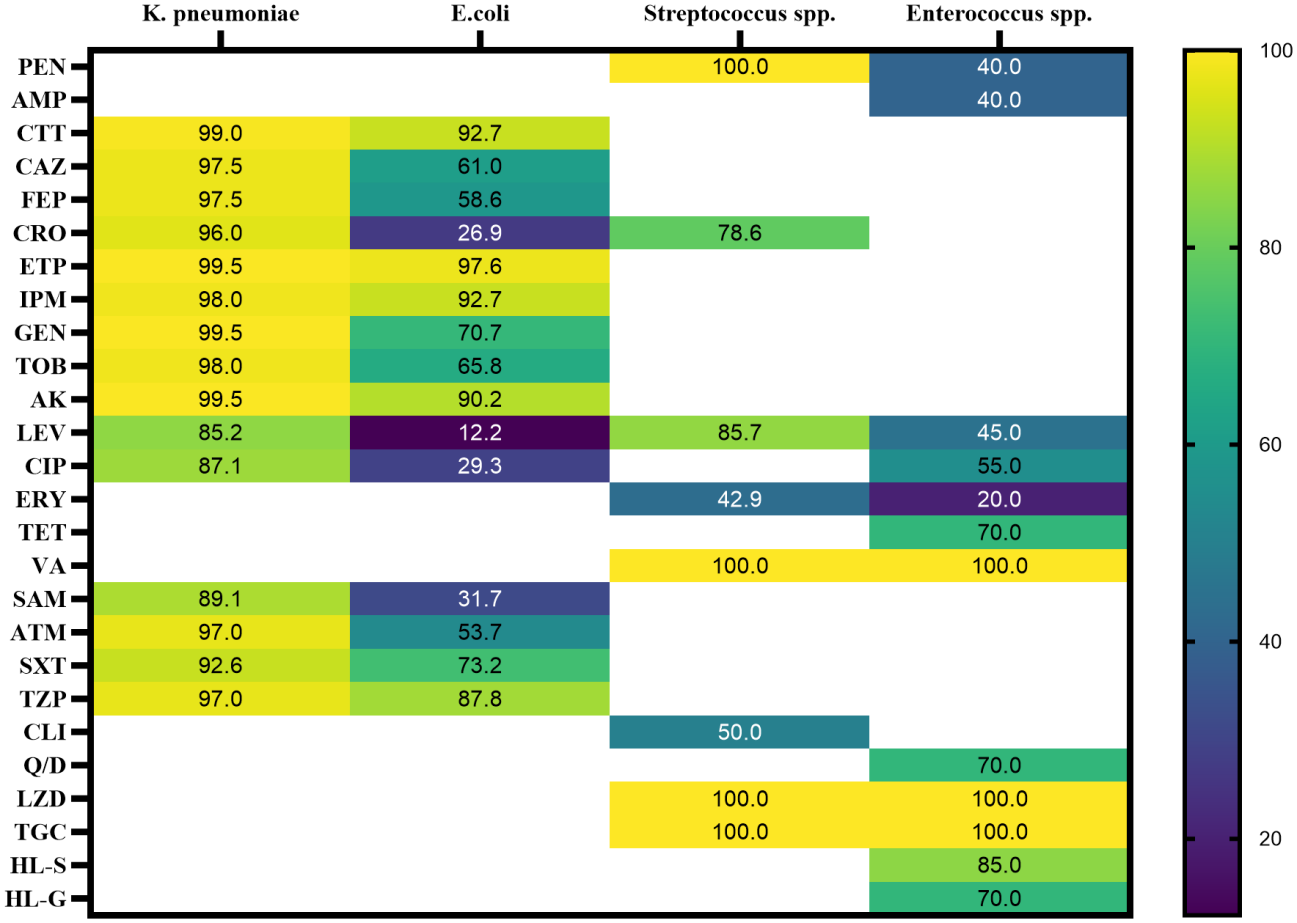
Heatmap of antibiotic susceptibility rates for four major pathogens isolated from patients with liver abscess. The heatmap displays the susceptibility rates of clinical isolates of *K. pneumoniae*, *E. coli*, *Streptococcus* spp., and *Enterococcus* spp. to a panel of antibiotics. The color gradient represents the percentage of susceptible isolates (ranging from low to high), as indicated by the color scale on the right. Values within each cell denote the proportion of susceptible strains (%).PEN, penicillin; AMP, ampicillin; CTT, cefotetan; CAZ, ceftazidime; FEP, cefepime; CRO, ceftriaxone; ETP, ertapenem; IPM, imipenem; GEN, gentamicin; TOB, tobramycin; AK, amikacin; LEV, levofloxacin; CIP, ciprofloxacin; ERY, erythromycin; TET, tetracycline; VA, vancomycin; SAM, ampicillin-sulbactam; ATM, aztreonam; SXT, trimethoprim-sulfamethoxazole; TZP, piperacillin-tazobactam; CLI, clindamycin; Q/D, quinupristin-dalfopristin; LZD, linezolid; TGC, tigecycline; HL-S, high-level streptomycin resistance; HL-G, high-level gentamicin resistance.

The top four pathogens isolated from liver abscesses were *K. pneumoniae*, *E. coli*, *Enterococcus* spp., and *Streptococcus* spp., and their susceptibilities to commonly used antibiotics are shown in **Figure 3**. Different pathogens displayed varying resistance patterns. *K. pneumoniae* and *E. coli* maintained high overall susceptibility to carbapenems, amikacin, and cefotetan, while *Enterococcus* spp. and *Streptococcus* spp. were highly susceptible to vancomycin, linezolid, and tigecycline. Notably, *E. coli* exhibited high resistance to quinolones and cefazolin, and *Enterococcus* spp also showed high resistance to penicillin and erythromycin.

**Figure 4:**
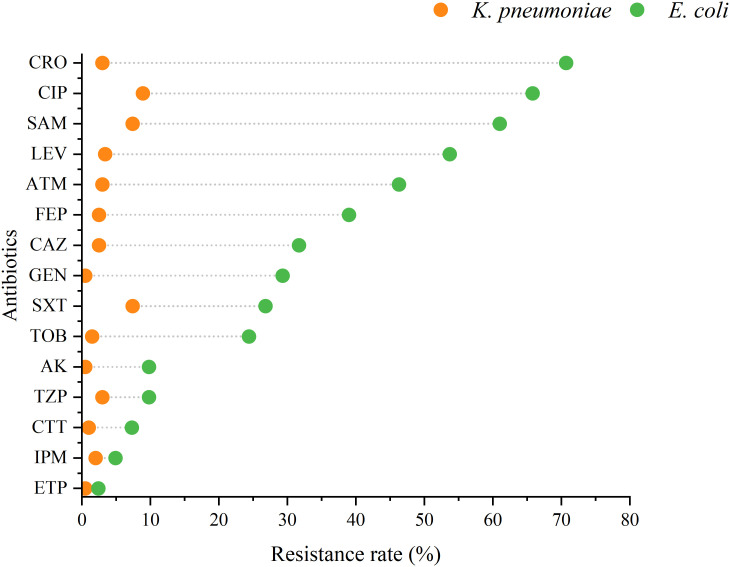
Comparative antimicrobial resistance patterns of *K. pneumoniae* and *E. coli* isolates.

Subsequently, we compared the resistance profiles of *K. pneumoniae* and *E. coli*, the two predominant Gram-negative pathogens in CPLA, as shown in **Figure 4**. K*. pneumoniae* exhibited a high susceptibility to most antimicrobial agents, whereas *E. coli* exhibited relatively higher resistance rates to fluoroquinolones, aztreonam, and cephalosporins. Both organisms maintained high susceptibility to carbapenems.

**Figure 5:**
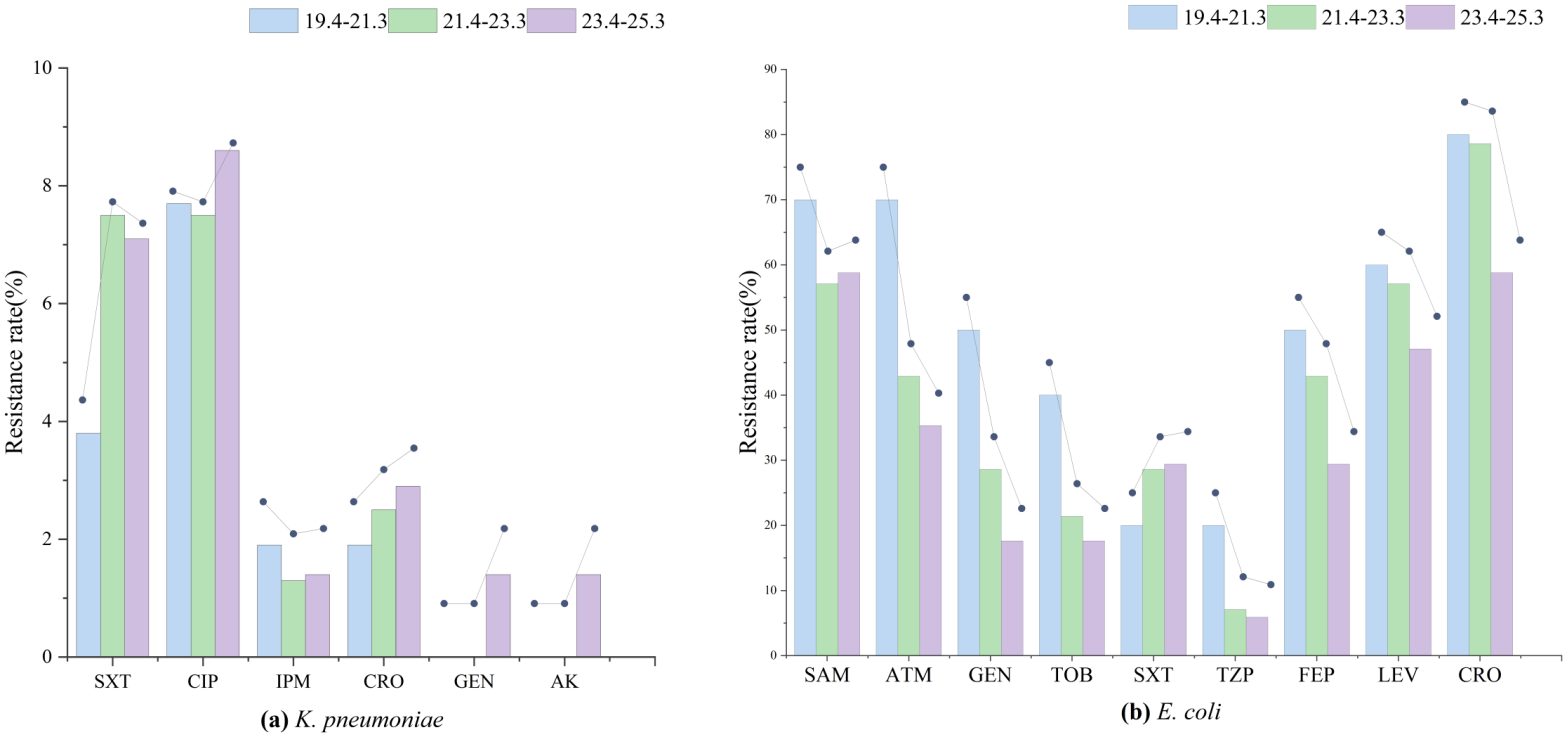
Temporal trends in antimicrobial resistance rates among *K. pneumoniae* and *E. coli* isolates during the study period. **(a)** Antimicrobial resistance trends of *K. pneumoniae*. **(b)** Antimicrobial resistance trends of *E. coli*. The Y-axis represents the resistance rate (%), defined as the percentage of isolates resistant to each antibiotic.

To further understand whether the antibiotic sensitivity profiles of these two pathogens have changed over time, we compared their resistance rates in three time periods (2019.4-2021.3 vs 2021.4-2023.3 vs 2023.4-2025.3), as shown in **Figure 5**. (*K. pneumoniae* showed an increasing resistance trend to Trimethoprim–sulfamethoxazole and Gentamicin, whereas resistance to Ciprofloxacin, Imipenem, and Ceftriaxone remained relatively stable. In contrast, *E. coli* exhibited a marked decrease in resistance to aztreonam, gentamicin, tobramycin, cefepime, and ceftriaxone, while resistance to trimethoprim–sulfamethoxazole and ertapenem showed a slight increase.

## 4 Discussion

In the present study, patients with CNLA accounted for 31.6% of the final cohort, indicating that culture-negative cases represent a substantial proportion of liver abscess patients in clinical practice. Compared with CNLA patients, patients with CPLA had longer hospital stays, more underlying comorbidities, and more severe clinical manifestations. These findings are consistent with previous studies (Yoon et al., 2019; Liu et al., 2023). Multivariate logistic regression analysis further demonstrated that diabetes, malignant tumors, and hepatic insufficiency were independently associated with culture positivity. One possible explanation is that patients with CPLA may have had more severe infections, leading to a higher incidence of sepsis and increased opportunities for pathogen detection in clinical specimens.

However, culture-negative status does not necessarily indicate a more favorable prognosis. A previous study (Shelat et al., 2016) has shown that patients with CNLA may still experience clinical outcomes comparable to those with culture-positive liver abscess, which is consistent with our findings. Although patients with CPLA had more severe clinical manifestations, pathogen identification may have facilitated subsequent pathogen-directed antimicrobial adjustment. In contrast, patients with CNLA still required empirical antimicrobial therapy in the absence of pathogen isolation, and culture negativity did not exclude clinically significant infection (Shelat et al., 2016; Yoon et al., 2019; Yoo et al., 2021).

Several factors may contribute to culture-negative results in liver abscess (Shelat et al., 2016; Serraino et al., 2018; Yoon et al., 2019; Li et al., 2021; Liu et al., 2023; Dudina et al., 2026). Antibiotic exposure before specimen collection is one of the most commonly recognized reasons, as antimicrobial therapy may reduce the bacterial burden in the bloodstream or abscess cavity, thereby decreasing the likelihood of pathogen recovery. In addition, blood cultures have limited sensitivity for identifying the causative pathogen of liver abscess, particularly when bacteremia is transient or absent. Some anaerobic or fastidious organisms may also be difficult to isolate under routine culture conditions. Therefore, culture-negative liver abscess does not necessarily indicate the absence of bacterial infection, but may instead reflect the combined effects of prior antimicrobial exposure, specimen source, bacterial burden, and limitations of conventional culture methods.

*K. pneumoniae* and *E. coli* were the most commonly isolated pathogens in patients with liver abscess, and notable differences in clinical characteristics were observed between the two groups. *K. pneumoniae* and *E. coli* were the most commonly isolated pathogens in patients with liver abscess, and notable differences in clinical characteristics were observed between the two groups. In this study, KPLA patients were relatively younger than ECLA patients and were more likely to have diabetes (Liu et al., 2024); previous studies have shown that type 2 diabetes mellitus predominantly affects individuals aged 40–65 years (Guo et al., ), whereas malignancies, which are more commonly associated with ECLA, predominantly occur in older populations, with a relatively higher incidence observed among individuals aged ≥60 years (Li et al., 2024), which may partly explain the observed age difference. Hyperglycemia can impair the phagocytic activity of neutrophils against K1/K2 *K. pneumoniae* liver abscess strains and enhance capsule-dependent evasion of the host immune system by bacteria, thereby facilitating the colonization and invasion of *K. pneumoniae* (Lam and Stokes, 2023; Wang et al., 2023). Chen’s (Chan et al., 2022) analysis revealed that *K. pneumoniae* liver abscess is highly correlated with the incidence of diabetes, while non-*K. pneumoniae* liver abscess is highly correlated with the incidence of liver and biliary tract diseases, as well as a history of malignant tumors (Klebsiella pneumoniae liver abscess: A new invasive syndrome, 2012). This is highly consistent with our research.

Several previous studies (Lee et al., 2008; Özşahin et al., 2026)have reported relatively high mortality rates in patients with LA, which may be associated with antimicrobial resistance, underlying comorbidities, and disease severity. In contrast, the overall mortality rate in our study was relatively low. This finding may be related to improvements in diagnostic techniques, antimicrobial therapy, image-guided drainage, and supportive management in recent years (Yoo et al., 2021). In addition, as a retrospective study, incomplete follow-up after discharge in some patients may also have influenced mortality assessment.

This study included a total of 297 patients with liver abscesses, and a total of 326 bacterial strains were isolated. The research showed that *K. pneumoniae* was the most frequently isolated strain in liver abscess infections, accounting for as high as 73.5%, followed by *E. coli*, accounting for 14.9%. Among the Gram-positive cocci, Enterococcus was the most common, especially *Enterococcus faecalis*, which was consistent with the previous research results (Xu et al., 2025). The slightly different detection rates of Gram-positive bacteria might be due to the sample size included and regional factors. In the past, *E. coli* was the most common pathogen causing liver abscesses. However, in recent years, *K. pneumoniae* has become the main pathogen causing such infections (Wang et al., 1998; Chung et al., 2007). Qin’s research also confirmed that the proportion of *K. pneumoniae* among the pathogenic bacteria causing liver abscesses has exceeded 60%, becoming the dominant pathogen (Long et al., 2024). Previous studies have suggested that the increasing predominance of *K. pneumoniae* in liver abscess may be partly associated with the emergence and spread of invasive *K. pneumoniae* strains, particularly those related to the K1 capsular serotype. However, the mechanisms underlying this epidemiological shift remain incompletely understood and require further investigation (Chuang et al., 2006; Chung et al., 2007; Yeh et al., 2007; Chung et al., 2008).

Due to the improper use of antibiotics, the resistance of bacteria is constantly increasing, and the proportion of multidrug-resistant bacteria is gradually rising. Our research shows that among 326 pathogenic bacteria, the proportion of multidrug-resistant bacteria is 22.4%. Among them, there are *E. coli* (n=28) and *K. pneumoniae* (n=13). The multidrug-resistance rate of *E. coli* and the rate of ESBL production are both very high, consistent with global monitoring data. However, in this study, the rate of ESBL-KP is lower, this difference may be related to variations in the number of included isolates and regional epidemiological characteristics (Karlowsky et al., 2022). Currently, carbapenem drugs remain effective treatments for ESBL-E, and the detection rate of carbapenem-resistant bacteria remains at a low level, with *K. pneumoniae* and *E. coli* being the main types.

Regarding the analysis of bacterial resistance in liver abscesses, the study found that *K. pneumoniae* has a relatively low overall resistance rate to commonly used antibiotics in clinical practice and maintains a high sensitivity to aminoglycosides and carbapenems. This is consistent with the results of many studies (Lee et al., 2017; Chen et al., 2022; Mai-Phan et al., 2026). However, drug-resistant strains of gentamicin and amikacin have emerged, and the resistance to trimethoprim–sulfamethoxazole has shown a significant increase. The emergence of aminoglycoside-resistant isolates may limit treatment options in selected patients and warrants clinical attention. Since trimethoprim–sulfamethoxazole is not commonly used as first-line therapy for KPLA, its increasing resistance is mainly relevant to local antimicrobial resistance surveillance. These findings highlight the need for continued monitoring of local antimicrobial susceptibility profiles. In contrast, the resistance situation of *E. coli* is more difficult. In this study, the resistance rate of *E. coli* to multiple antibiotics was significantly higher than that of *K. pneumoniae*, especially ceftriaxone, ampicillin-sulbactam, and fluoroquinolones. This difference in antimicrobial resistance may be related to the different resistance mechanisms and carrying rates of resistance genes of the two bacteria. As a common colonizing bacterium in the intestine, *E. coli* is exposed to various antibiotics for a long time, making it more likely to acquire and spread resistance genes (Sapkota et al., 2021).

These findings have practical implications for empirical antimicrobial therapy and clinical management. For patients without microbiological evidence or with culture-negative results, empirical therapy should prioritize coverage of Enterobacterales. On this basis, if the patient has concomitant diabetes, *K. pneumoniae* infection should be further suspected; if concomitant malignancy is present, *E. coli* infection should be suspected. Given the higher antimicrobial resistance burden observed in *E. coli*, susceptibility-guided therapeutic adjustment should be emphasized for such infections, with treatment optimization based on subsequent culture and susceptibility results (Liu et al., 2021).

In summary, this study describes the distribution of pathogenic bacteria and the drug resistance spectrum of liver abscesses, compares the drug resistance patterns of the two major pathogenic bacteria of liver abscesses – *K. pneumoniae* and *E. coli*, and comparative analyses were performed from both culture -based (CPLA vs. CNLA) and pathogen-based(KPLA vs. ECLA) perspectives.

However, this study has several limitations. First, this was a single-center retrospective study, which may have introduced selection bias and limited the generalizability of the findings. Second, some detailed clinical information was not available in the retrospective dataset, including abscess imaging characteristics, timing of drainage interventions, antimicrobial exposure before specimen collection, timing of culture, and antimicrobial adjustment processes. Therefore, we could not further evaluate the specific effects of these factors on culture results, treatment decisions, or clinical outcomes. Third, long-term outcomes, such as recurrence, readmission, and post-discharge mortality, were not collected, and some patients experienced transfer to other departments, transfer to other hospitals, or discharge against medical advice, which may have affected the accuracy of outcome assessment. In addition, because antimicrobial-resistant isolates and adverse outcome events were relatively limited, further resistance-based outcome analyses were not performed.

## 5 Conclusions

In summary, there are significant differences in clinical characteristics between CPLA and CNLA, with a substantial proportion of cases being CNLA, which to some extent limits the implementation of precise antimicrobial therapy. *K. pneumoniae* and *E. coli* remain the predominant pathogens; however, they differ in clinical characteristics and antimicrobial resistance profiles. In this cohort, KPLA was more frequently associated with diabetes and generally remained susceptible to commonly used antimicrobials, whereas ECLA was more commonly associated with malignant tumors and biliary tract disease and demonstrated a higher burden of antimicrobial resistance. These findings emphasize that the clinical management of liver abscess should integrate underlying comorbidities, pathogen distribution, and resistance patterns. In CNLA or when microbiological results are not yet available, empirical antimicrobial therapy should cover common Enterobacterales, followed by timely adjustment based on microbiological and susceptibility results. Given the higher resistance burden observed in ECLA, clinicians should place greater emphasis on susceptibility-guided treatment adjustment in patients with *E. coli* infection to achieve timely optimization and effective clinical management.

## Data Availability

The raw data supporting the conclusions of this article will be made available by the authors, without undue reservation.
